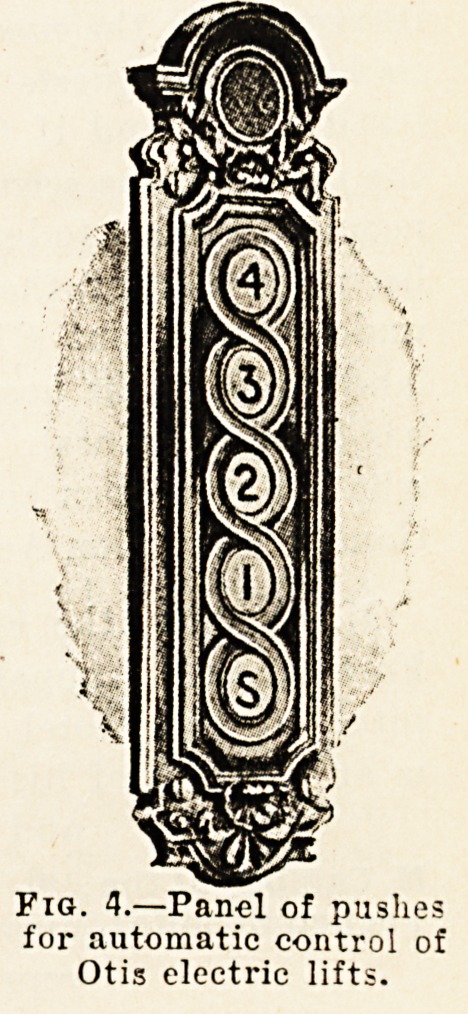# Electric Lifts for Hospital Use

**Published:** 1910-05-07

**Authors:** 


### ELECTRIC LIFTS FOR HOSPITAL USE.

A lift of any form is a great boon in hospital work. In fact it is hardly too much to say that the work of the modern hospital cannot be properly carried on without the aid of lifts. The old-time lift is sadly out of place, and in these days the jolting that was its accompaniment would hardly be tolerated. The hydraulic lift was a great improvement upon the earlier form, in which a simple rope and winch were employed, but even the hydraulic lift is hardly smooth enough for the requirements of a busy modern hospital. Those who have used the hydraulic lift will remember that there is a nasty jerk when the attendant pulls the rope which opens the valve allowing the lift to move, and there is another nasty jerk when the lift is brought to rest. In the electric lift, in its latest form, all of that has been done away with. Further, electricity enables the attendant to be dispensed with; the movement of the lift itself is controlled from each floor, or from the inside of the lift.

In the modern electric lift—the car, guides, and supporting pulley of those made by the Otis Company are shown in fig. 1—the movement of the lift is accomplished by means of wire ropes passing over pulleys at the head of the well and wound on and off a drum driven by an electric motor. The drum and motor may be placed at the bottom of the well, or at the top, as convenient. The car, or cage as it is sometimes called, slides up and down the well provided for it, supported by the rope mentioned and held between either wooden or steel guides, steel being more common in modern buildings. The control of the motion of the car is obtained by means of the apparatus shown in fig. 2. Those who have used electric motors will remember that there is always a starting switch, arranged to allow current to pass into the motor by degrees, the handle of the switch moving a contact bar over different stops. In the electric lift, when it is controlled entirely from the car, the apparatus shown in fig. 3 is fixed on the car, and the attendant causes the lift to move up or down by moving a handle which is slipped over the projecting pin, shown at the top of the figure, to the right or left. The motion of the handle makes certain contacts within the apparatus, which in their turn causes a series of electromagnets to come into operation; the result is that the car moves off very gently, and is brought very gently to rest.

A later development of this, which is employed more generally in hospitals, is what is known as the push-button automatic control. With this arrangement there is a push fixed outside of the well at each landing, and there is also a series of pushes on the car, as shown in fig. 4, one for each landing and one for stopping in case of emergency—*i.e.* in case, after the car has been started, it is desired to stop it to change its destination or direction of travel. In the illustration pushes for four landings and one stopping push are shown. In operation, when the

Fig. 1.—Showing the general arrangement of Otis electric lift, with car, guides, ropes, &c. The governor, which brings the safety catch into operation, is seen at the top, on the left, small.

lift is wanted at any landing, whoever requires it presses the push on the outside of the well and waits till the car comes to that landing. If the car is in use at the time the pushes on the landings are inoperative until those who are in possession of the car have done with it. Further, it is

Fig. 2.—Controller of Otis electric automatic lift arranged that the gates of the well at the different landings cannot be opened while the car is in motion, nor until it has been brought completely to rest, its passengers discharged, and the gate closed. This again is a most important provision for safety, bearing in mind the accidents that have occasionally occurred from persons falling down lift wells. It will be seen that by the push-button automatic control the necessity for an attendant is completely dispensed with.

### Safety Arrangements.

Careful provision is made for safety of passengers by the lift by the following arrangement. In the Otis Company's lifts, from which our illustrations are drawn, the motor and the winding-drum, which give motion to the car, are strongly braked when the car is at rest, so that it is impossible to move it until either the car handle or one of the push-buttons has been moved. The first operation when the lift is to be set in motion is the removal of the pressure of the brake by a special electro-matgnet. If anything happens to the service, if the current is cut off, or if there is any failure, the brake at the motor immediately comes into operation. In addition to this, there are safeties on the car for arresting its descent if a lifting rope breaks or stretches unduly; there is also a governor, shown very small at the top on the left of fig. 1, which comes into operation and applies the car safeties, whether ropes are broken or intact, should the speed of the car

Fig. 3.—Car-switch of Otis electric lift.

Fig. 4.—Panel of pushes for automatic control of Otis electric lifts.

under any circumstances exceed a certain limit. By further arrangements the guides are held to the shoes upon the car should anything happen to the hoisting arrangements. Should anything happen to any part of the arrangement for raising and lowering the car, the latter is immediately and gently brought to rest, at whatever point it may be. It can then be raised gently to the nearest landing, the passengers get out, and the trouble is made good.

## Figures and Tables

**Fig. 1. f1:**
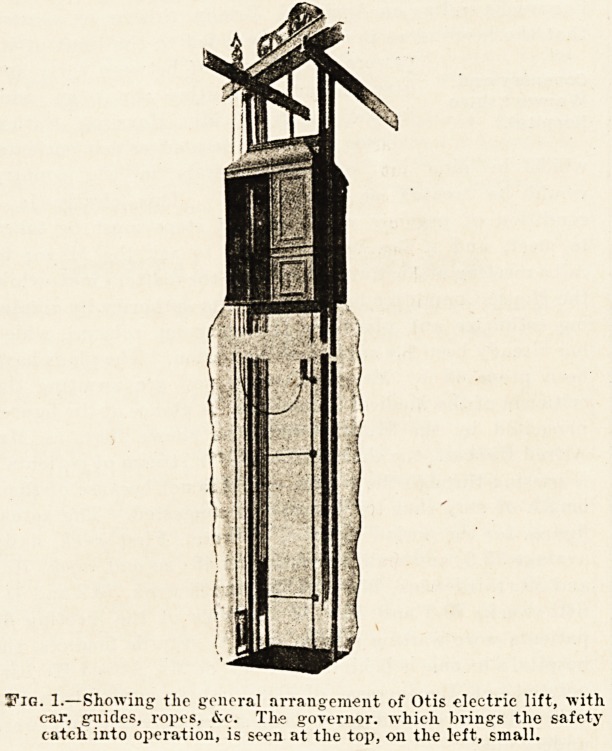


**Fig. 2. f2:**
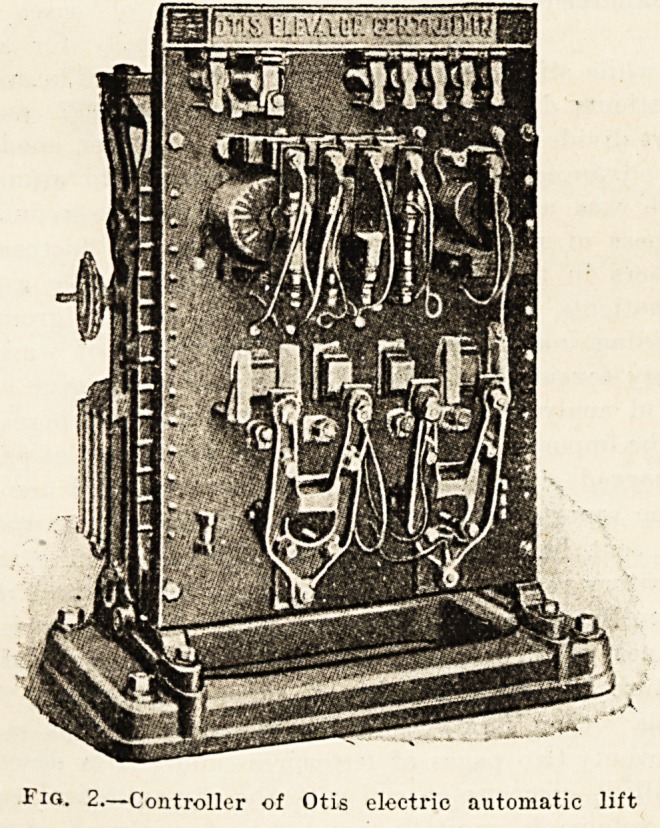


**Fig. 3. f3:**
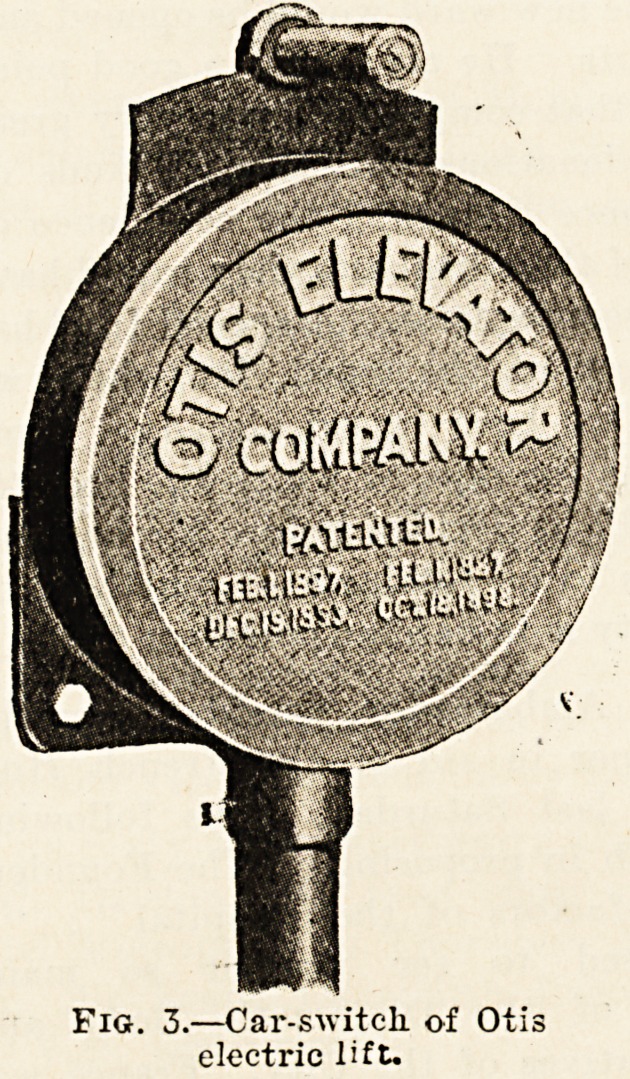


**Fig. 4. f4:**